# Risk of Acute Myocardial Infarction in Pneumoconiosis: Results from a Retrospective Cohort Study

**DOI:** 10.3390/biomedicines11030897

**Published:** 2023-03-14

**Authors:** Ju-Hsin Chang, Te-Chun Shen, Ke-Wei Chen, Cheng-Li Lin, Chung Y. Hsu, Yeong-Ray Wen, Kuan-Cheng Chang

**Affiliations:** 1 Graduate Institute of Clinical Medicine Science, China Medical University, No. 91, Xue-Shi Road, Taichung 404, Taiwan; 2 Department of Anesthesia, China Medical University Hospital, Taichung 404, Taiwan; 3 Division of Pulmonary and Critical Care Medicine, Department of Internal Medicine, China Medical University Hospital, Taichung 404, Taiwan; 4 School of Medicine, China Medical University, Taichung 404, Taiwan; 5 Department of Critical Care Medicine, Chu Shang Show Chwan Hospital, Nantou 557, Taiwan; 6 Division of Cardiology, Department of Internal Medicine, China Medical University Hospital, No. 2 Yu-De Road, Taichung 404, Taiwan; 7 Management Office for Health Data, China Medical University Hospital, Taichung 404, Taiwan

**Keywords:** interstitial lung disease (ILD), pneumoconiosis, coronary artery disease (CAD), acute myocardial infarction (AMI)

## Abstract

Background: Pneumoconiosis (PCN) has several comorbidities, most notably pulmonary and cardiovascular diseases. However, much is still unknown about the relationship between PCN and acute myocardial infarction (AMI). The present study aimed to clarify the association between PCN and subsequent AMI risk using a retrospective cohort study design. Methods: This was a population-based, retrospective cohort study that used data from Taiwan’s National Health Insurance Database. A total of 7556 newly diagnosed patients with PCN and 7556 individuals without PCN were included in the PCN and comparison cohort (PC and CC), respectively, between 2008 and 2018, with propensity score matching for age, gender, comorbidity, medication, and date of PCN diagnosis. The occurrence of AMI was monitored until the end of 2019, and AMI risk was assessed using Cox proportional hazard regression models. Results: The overall incidence of AMI was 1.34-fold higher in the PC than in the CC (4.33 vs. 3.23 per 1000 person-years, respectively, *p* < 0.05), with an adjusted hazard ratio (aHR) of 1.36 (95% confidence interval (CI): 1.08–1.72) after controlling for age, gender, comorbidity, and medication. Further analyses showed a higher risk of AMI with increased annual number of emergency department visits among patients with PCN (aHR: 1.30, 95% CI: 1.01–1.66 (<1) and aHR: 1.68, 95% CI: 1.13–2.50 (≥1)). Conclusion: Patients with PCN had a significantly higher risk of developing AMI than those without PCN. Clinicians should pay more attention to prevent AMI episodes in patients with PCN.

## 1. Introduction

Pneumoconiosis (PCN) is one of a group of interstitial lung diseases caused by inhalation of certain kinds of dust particles that damage the lungs [1]. PCN can be simple or complicated. Simple PCN causes small and round nodules and complicated PCN causes a lot of scarring and fibrosis tissues in the lungs. Prevalence and incidence of PCN have remained high in recent decades worldwide. There are predicted to be 500,000 alive cases and 60,000 new cases annually. Mortality from PCN also remains high, with more than 20,000 deaths annually [2]. Various kinds of mineral dust could cause various respiratory and cardiovascular diseases. PCN is associated with poor prognosis from these diseases [3,4,5].

Acute myocardial infarction (AMI) is a medical emergency caused by gross cell death of the cardiac muscle as a result of ischemia, often diagnosed clinically through the cardinal symptoms and signs, electrocardiography (ECG), biochemical testing, non-invasive and invasive imaging techniques, and consecutive pathological evaluation. Approximately 550,000 first episodes and 200,000 recurrent episodes of AMI occur each year in the United States [6]. An atherosclerotic patch dislodges to form coronary plaque, which can compromise the blood vessel patency leading to AMI, which has a high mortality rate. Severe types of AMI are predominantly identified as ST-segment elevation in ECG. Lifestyle, environmental, and genetic factors are contributory factors for AMI. Common risk factors include smoking, diabetes mellitus (DM), hyperlipidemia (HL), and hypertension (HTN). Obesity, homocystinuria, hyperuricemia, and psychosocial stress are also risk factors [7]. However, many potential risk factors are still under investigation.

Several pulmonary and cardiovascular comorbidities have been considered associated with PCN [8,9,10,11,12]. Previously investigated comorbidities included HTN, DM, HL, ischemic heart disease, atrial fibrillation, congestive heart failure, chronic obstructive pulmonary disease (COPD), gastroesophageal reflux disease, and chronic kidney disease (CKD) [3,13]. Most previous studies on the subsequent effects of PCN have focused on interstitial lung disease, pulmonary fibrosis, COPD, and lung cancer. However, blood vessel injuries related to PCN have not been often researched and documented. Using multidetector computed tomography, Lee et al. [14] analyzed the coronary artery calcification (CAC) among 76 patients who were exposed to inorganic dust and reported that PCN was more common among patients with CAC than the non-CAC population. They suggested that additional research is imperative to evaluate the inflammatory reaction of PCN and its influence on atherosclerosis and coronary artery disease (CAD). Since AMI is an important but largely unexplored medical condition, the current study aimed to explore the relationship between PCN and AMI risk.

## 2. Materials and Methods

### 2.1. Data Source

**Figure 1:**
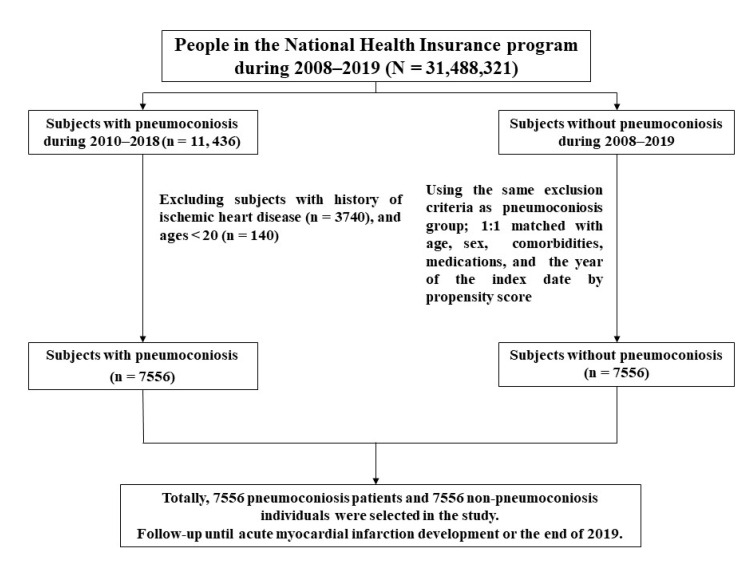
Flowchart of participant selection.

Taiwan introduced a single-payer National Health Insurance (NHI) program on 1 March 1995, in which 99.9% of the population has enrolled since 2014. Here, a database comprising the registration files and original claims data of all individuals regarding reimbursement was organized. Many databases resulting from this database are provided to scientists in Taiwan for research activities. Data in the NHI can be utilized for locating patients or healthcare providers, such as medical organizations, institutions, and physicians; however, these data were jumbled before being handed over to researchers. Thus, it is theoretically impossible to query the data alone to identify individuals at any level using this database. Citizens of Taiwan who were eligible to research were alone entitled to apply to this database which was exclusively for research use. Applicants must follow the Computer-processed Personal Data Protection Law and associated regulations of Taiwan’s Ministry of Health and Welfare, and an agreement must be signed by the applicant and his/her supervisor upon application submission. All applications are reviewed before data are released. Each year, Taiwan’s Ministry of Health and Welfare gathers information from the NHI database and sorts them as data files, including registration files and original claims data for reimbursement. These data files are de-coded by jumbling the identification codes of patients and medical facilities and constructing original files of the NHI database. In this study, we used the NHI database, which included 31,488,321 individuals and detailed medical information from 2008 to 2019 (Figure 1). This study was approved by the Research Ethics Committee of China Medical University Hospital (CMUH110-REC3-133).

### 2.2. Study Cohorts

The PCN cohort (PC) enrolled newly diagnosed adult patients between 2008 and 2018 (International Classification of Diseases (ICD) codes 500, 501, 502, 503, 505, J60, J61, J62, J63, and J64). In the present study, the common cause of PCN was inhalation of mineral dust. Mineral dust includes coal dust, asbestos, crystalline silica, and other materials. The detailed codes were coal worker’s PCN (ICD code 500 and J60), PCN due to asbestos (ICD code 501 and J61), PCN due to dust containing silica (ICD code 502 and J62), PCN due to other inorganic dusts (ICD code 503 and J63), and unspecified PCN (ICD code 505 and J64). There were some other references using similar definitions [11,15]. We excluded those patients having ischemic heart disease (ICD codes 410–414 and I20–I25) before the diagnosis of PCN. We enrolled participants free of PCN in the comparison cohort (CC), after propensity score matching for age, gender, comorbidity, medication, and date of PCN diagnosis (index date). Individuals who had ischemic heart disease prior to the index date were also excluded. Both cohorts were followed until an AMI episode, withdrawal from insurance system, death, or 31 December 2019.

### 2.3. Outcome and Related Factors

The primary outcome was AMI (ICD codes 410 and I21), and age, gender, comorbidities, and medication were confounding variables. Hence, various associated comorbidities at baseline, such as HTN (ICD codes 401–405 and I10–I16), DM (ICD codes 250 and E08–E13), HL (ICD codes 272 and E78), asthma/COPD (ICD codes 491, 492, 493, 496, and J41–J45), cerebrovascular disease (CVD, ICD 430–438 and I60–I69), and CKD (ICD 585 and N18) were identified for adjustment. Corticosteroid users were defined as those who used the medication for more than 28 days.

### 2.4. Statistical Analysis

The proportions of age distribution, gender, comorbidity, and medication in both groups were compared using the Chi-squared test. The mean age was analyzed through Student’s *t*-test. The cumulative incidences of AMI in the PC and CC were calculated using the Kaplan–Meier test, and the log-rank test was employed to determine the significance level. Univariable and multivariable Cox proportional hazard regression models were employed to evaluate crude and adjusted hazard ratios (cHRs and aHRs) with 95% confidence intervals (CIs). Data were analyzed using the SAS statistical software (version 9.4 for Windows; SAS Institute, Inc., Cary, NC, USA). A *p*-value < 0.05 was considered statistically significant.

## 3. Results

**Figure 2:**
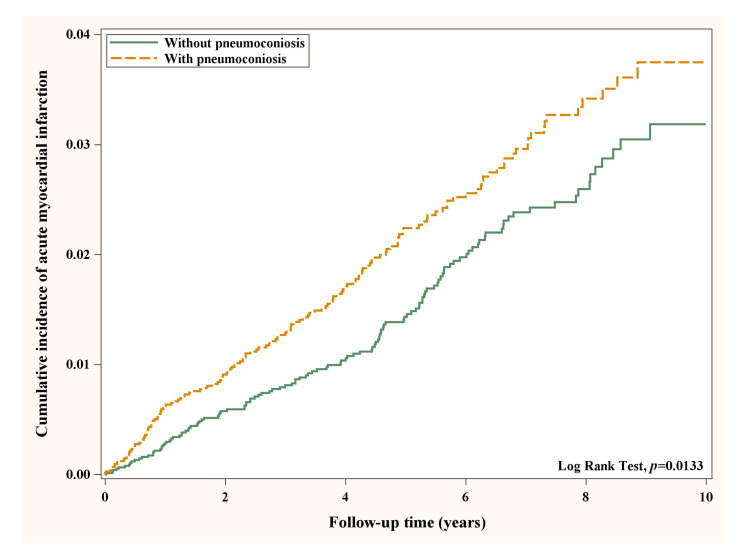
Cumulative incidence of acute myocardial infarction in the pneumoconiosis cohort (PC) and comparison cohort (CC).

There was a total of 7556 individuals in the PC and 7556 individuals in the CC (Table 1). The proportions of coal worker’s PCN, PCN due to dust containing silica, PCN due to asbestos, and PCN due to other or unspecified dust were 38.3%, 4.7%, 1.5%, and 55.5%, respectively. The mean age was 66.9 ± 13.1 years in the PC and 66.8 ± 13.3 years in the CC. Both cohorts consisted of 85.8% males. The extents of comorbidities and medication in the PC and CC were as follows: HTN (43.6% vs. 44.0%), DM (17.9% vs. 18.4%), HL (23.9% vs. 24.3%), asthma/COPD (44.3% vs. 44.9%), CVD (11.56% vs. 9.23%), CKD (4.55% vs. 4.98%), and corticosteroid use (45.3% vs. 45.3%). There was no significant difference in age, gender, comorbidities, and corticosteroid usage between the PC and CC. The average follow-up time was 4.74 ± 2.87 and 5.25 ± 2.73 years in the PC and CC, respectively, during which the increasing incidence of AMI was remarkably greater in the PC than in the CC (Figure 2, *p* = 0.0133 in the log-rank test).

The overall occurrence of AMI was 1.34 times greater in the PC than in the CC (4.33 vs. 3.23 per 1000 person-years, respectively, *p* < 0.05, Table 2), with an aHR of 1.36 (95% CI: 1.08–1.72) following controlling for age, gender, comorbidity, and medication usage. The aHRs of AMI were 2.95 times greater in patients aged between 50–64 years (95% CI: 1.35–6.44) and 4.40 times greater in those aged 65 years and above (95% CI: 2.05–9.45) than in those aged between 20–49 years. The aHR of AMI was 2.84 times greater in males than in females (95% CI: 1.71–4.72). Further, the AMI risk was remarkably higher in participants with HTN (aHR: 1.63, 95% CI: 1.25–2.11) and DM (aHR: 1.57, 95% CI: 1.17–2.10) than in those without. The incidence rates of AMI were also greater among patients with HL, asthma/COPD, CVD, and CKD than those without, but without statistical significance.

Table 3 shows the incidence and aHR of AMI for the PC and CC by age, gender, and presence of comorbidities. In the <65 and ≥65 years age groups, the age-specific aHRs in the PC compared to the CC were 1.55 (95% CI: 0.98–2.43) and 1.32 (95% CI: 1.00–1.73), respectively. The gender-specific aHRs in the PC compared to the CC were 1.06 (95% CI: 0.40–2.82) in females and 1.38 (95% CI: 1.08–1.76) in males. The comorbidity-specific aHRs in the PC compared to the CC were 1.10 (95% CI: 0.85–1.42) in participants presenting with comorbidity and 3.99 (95% CI: 2.09–7.61) in participants without comorbidity.

Table 4 shows that AMI risk was further higher for participants that had increased annual emergency medical demands in the PC compared with CC (aHR: 1.30, 95% CI: 1.01–1.66 (<1) and aHR: 1.68, 95% CI: 1.13–2.50 (≥1)).

## 4. Discussion

In this population-based, retrospective cohort study that investigated the association between PCN and risk factors for AMI, subjects with PCN had a greater risk of AMI compared to non-PCN subjects. The incidence of AMI was similar between PCN patients with and without any comorbidity. Additionally, the AMI risk was greater for patients with PCN who had proportionally greater frequency of annual emergency department visits. Finally, this cohort highlighted useful epidemiologic data for PCN on a large scale.

The prevalence of CAD among PCN has been presented by Paul et al., who collected information from 8531 patients with PCN from the 5% Medicare Claims Limited Data Set in the United States between 2011 and 2014 [5]. They reported that CAD occurred in 13.9% of all patients with PCN and accounted for 18.7% of those who lost their lives. In their study, Beggs et al. reported patterns of PCN mortality in Kentucky [4]. Of the 330 deaths with PCN, there was a large proportion of heart-related deaths (23.3%), with 37 (12.2%) from cardiac arrest, 16 (4.8%) from myocardial infarction, 16 (4.8%) from congestive heart failure, and 8 (2.4%) from arteriosclerosis. In addition, Hu et al. enrolled 12,209 patients with PCN from the Taiwan NHI database and registered that 2919 (23.9%) of them had CAD [11]. This finding indicates that CAD is a significant comorbidity and cause of death among patients with PCN.

The patho-mechanical connection between PCN and atherosclerosis was illustrated, starting from dust inhalation that triggers a prothrombotic activity via the interleukin-6-dependent system, leading to diminished clotting time, intravascular thrombin formation, and accelerated arterial thrombosis [16]. In addition, exposure to dust particles could increase plasminogen activator inhibitor-1 and suppress tissue plasminogen activator, causing impaired fibrinolysis [17]. Moreover, dust exposure could suppress the tissue factor pathway inhibitor and enhance the extrinsic coagulation pathway to exacerbate intravascular thrombosis [18]. Overall, PCN-related inflammatory reaction, blood vessel injury, and thrombosis-embolic activity might play major roles in the formation of atherosclerosis and following AMI [19,20].

It is crucial to understand the differences in cardiovascular comorbidities in people with and without PCN, which is pivotal in the development of AMI. In the study, we performed propensity score matching for the comorbidities, and thus, no difference was observed for all comorbidities between the PCN group and the comparison group. However, in several studies that did not match for comorbidities, we can find the difference in cardiovascular comorbidities between people with and without PCN. Shen et al. [8] collected data of 3374 patients with PCN from the Registry of Catastrophic Illness database and 13,496 individuals without PCN from the Longitudinal Health Insurance Database in Taiwan. They found that the magnitudes of cardiovascular comorbidities in the PC and CC were as follows: HTN (48.96% vs. 48.47%, *p* = 0.62), DM (13.63% vs. 16.28%, *p* < 0.001), HL (20.45% vs. 21.83%, *p* = 0.09), asthma (30.32% vs. 7.48%, *p* < 0.001), COPD (61.65% vs. 5.42%, *p* < 0.001), heart failure (6.85% vs. 3.81%, *p* < 0.001), CVD (6.37% vs. 5.79%, *p* = 0.22), and CKD (1.72% vs. 1.99%, *p* = 0.35). Cheng et al. [9] established a PC (*n* = 1238) from the Registry of Catastrophic Illness database and a CC (*n* = 4952) from the Longitudinal Health Insurance Database; they found that the magnitudes of cardiovascular comorbidities in the PC and CC were as follows: HTN (25.2% vs. 23.8%, *p* = 0.305), DM (10.1% vs. 11.3%, *p* = 0.217), HL (10.3% vs. 8.4%, *p* = 0.039), COPD (25.0% vs. 11.1%, *p* < 0.001), and CKD (0.6% vs. 1.2%, *p* = 0.119). Chuang et al. [10] obtained data of 6940 patients with PCN from the Registry of Catastrophic Illness database and 27,760 individuals without PCN from the Longitudinal Health Insurance Database; they found that the proportions of cardiovascular comorbidities in the PC and CC were as follows: HTN (34.5% vs. 34.3%, *p* = 0.84), DM (4.97% vs. 6.45%, *p* < 0.001), HL (11.4% vs. 13.7%, *p* < 0.001), COPD (50.6% vs. 8.79%, *p* < 0.001), heart failure (1.76% vs. 0.79%, *p* < 0.001), and atrial fibrillation (0.43% vs. 0.34%, *p* = 0.24). Yen et al. [12] enrolled 8923 patients with PCN from the Registry of Catastrophic Illness database and 35,692 individuals without PCN from the Longitudinal Health Insurance Database; they noted that the proportions of cardiovascular comorbidities in the PC and CC were as follows: HTN (42.3% vs. 44.9%, *p* < 0.001), DM (6.52% vs. 8.79%, *p* < 0.001), HL (15.8% vs. 18.4%, *p* < 0.001), COPD (52.4% vs. 11.7%, *p* < 0.001), and CVD (4.81% vs. 5.32%, *p* = 0.05). In the comorbidity analyses in these studies, we found that HTN, DM, and HL had heterogeneous differences between those with and without PCN when in different study designs; COPD and heart failure had persistent predominantly higher prevalence in patients with PCN than those without PCN. Additionally, the present study used the entire population (*n* = 31,488,321) in the same NHI database to identify those with and without PCN, and it was believed to have more precise results.

The large PC and absolutely matched cohort used for comparison in this study should be rated as the first strength of this retrospective cohort study. Almost every subject accomplished the follow-up. A prospective cohort study might be expensive, hence, was not feasible; thus, an alternative less expensive retrospective cohort study was preferred utilizing Taiwan’s NHI database [21,22,23]. In addition, this cohort reflected a “real world” scenario in which PCN, AMI, and all comorbidities were clinically and physically assessed during medical consultation.

The operational definitions for PCN, AMI, and comorbidities were adopted from ICD codes, and all diagnoses were dependent on the skill and competence level of clinicians. If this factor had influenced the study results confoundingly, that might be considered a limitation. Detailed information on weight, body mass index, history of smoking, occupational hazard exposure, and family history were lacking in the database. This is also a limitation, as all of them are relevant risk factors for AMI. Third, related clinical variables such as laboratory data (cardiac enzymes, b-type natriuretic peptide, glycated hemoglobin, hemoglobin, etc.), pulmonary function tests, ECG, echocardiography, chest radiography and computed tomography results, and pathology findings were unavailable [15].

## 5. Conclusions

Patients with PCN had a significantly greater risk for AMI compared to subjects without PCN. The incidence of AMI was similar between PCN patients with and without any comorbidity. Clinicians should pay attention to prevent AMI episodes in patients with PCN, even in those without obvious cardiovascular comorbidity.

## Figures and Tables

**Table 1:** Baseline characteristics in the study population.

	Pneumoconiosis				
	No		Yes		
	*N* = 7556		*N* = 7556		
	*n*	%	*n*	%	*p*-Value ^†^
Age					0.89
20–49	738	9.8	725	9.6	
50–64	2302	30.5	2324	30.8	
≥65	4516	59.8	4507	59.7	
Mean ± SD	66.8	±13.3	66.9	±13.1	0.70
Gender					0.96
Women	1070	14.2	1072	14.2	
Men	6486	85.8	6484	85.8	
Comorbidity					
HTN	3327	44.0	3297	43.6	0.62
DM	1392	18.4	1350	17.9	0.38
HL	1837	24.3	1806	23.9	0.56
Asthma/COPD	3391	44.9	3348	44.3	0.48
CVD	1055	14.0	1035	13.7	0.64
CKD	376	4.98	344	4.55	0.22
Medication					1.00
Corticosteroid	3425	45.3	3425	45.3	

CKD = chronic kidney disease; COPD = chronic obstructive pulmonary disease; CVD = cerebrovascular disease; DM = diabetes mellitus; HL = hyperlipidemia; HTN = hypertension; SD = standard deviation. ^†^ Chi-squared test and *t*-test.

**Table 2:** Risk factors of acute myocardial infarction among the study participants.

	Event	PY	IR ^†^	Crude HR (95% CI)	Adjusted HR ^#^ (95% CI)
Pneumoconiosis					
No	128	39,675	3.23	1 (Reference)	1 (Reference)
Yes	155	35,799	4.33	1.34 (1.06–1.70) *	1.36 (1.08–1.72) *
Age					
20–49	7	8418	0.83	1 (Reference)	1 (Reference)
50–64	70	24,990	2.80	3.36 (1.55–7.31) **	2.95 (1.35–6.44) **
≥65	206	42,066	4.90	5.90 (2.78–12.5) ***	4.40 (2.05–9.45) ***
Gender					
Women	16	11,136	1.44	1 (Reference)	1 (Reference)
Men	267	64,339	4.15	2.89 (1.75–4.79) ***	2.84 (1.71–4.72) ***
Comorbidity					
HTN					
No	117	44,428	2.63	1 (Reference)	1 (Reference)
Yes	166	31,046	5.35	2.04 (1.61–2.58) ***	1.63 (1.25–2.11) ***
DM					
No	209	63,228	3.31	1 (Reference)	1 (Reference)
Yes	74	12,246	6.04	1.84 (1.41–2.39) ***	1.57 (1.17–2.10) **
HL					
No	206	57,784	3.57	1 (Reference)	1 (Reference)
Yes	77	17,690	4.35	1.22 (0.94–1.59)	0.93 (0.70–1.25)
Asthma/COPD					
No	148	44,336	3.34	1 (Reference)	1 (Reference)
Yes	135	31,138	4.34	1.30 (1.03–1.65) *	1.22 (0.96–1.55)
CVD					
No	241	67,118	3.59	1 (Reference)	1 (Reference)
Yes	42	8356	5.03	1.41 (1.01–1.95) *	0.94 (0.67–1.32)
CKD					
No	263	72,743	3.62	1 (Reference)	1 (Reference)
Yes	20	2731	7.32	2.04 (1.30–3.22) **	1.51 (0.95–2.41)

CI = confidence interval; CKD = chronic kidney disease; COPD = chronic obstructive pulmonary disease; CVD = cerebrovascular disease; DM = diabetes mellitus; HL = hyperlipidemia; HR = hazard ratio; HTN = hypertension; IR = incidence rate; PY = person-years. ^†^ Incidence rate per 1000 person-years. ^#^ Adjustment for age, gender, comorbidity, and medication; * *p* < 0.05, ** *p* < 0.01, *** *p* < 0.001.

**Table 3:** Incidence rates and hazard ratios of acute myocardial infarction for the pneumoconiosis cohort (PC) compared to the comparison cohort (CC) by age, gender, and comorbidity.

	Pneumoconiosis							
	No			Yes				
	Event	PY	IR ^†^	Event	PY	IR ^†^	Crude HR (95% CI)	Adjusted HR ^#^ (95% CI)
Age								
<65	32	17,204	1.86	45	16,203	2.78	1.50 (0.95–2.36)	1.55 (0.98–2.43)
≥65	96	22,470	4.27	110	19,596	5.61	1.32 (1.00–1.73) *	1.32 (1.00–1.73) *
Gender								
Women	8	5673	1.41	8	5463	1.46	1.03 (0.39–2.75)	1.06 (0.40–2.82)
Men	120	34,002	3.53	147	30,336	4.85	1.38 (1.08–1.75) **	1.38 (1.08–1.76) **
Comorbidity ^‡^								
No	12	10,999	1.09	40	9944	4.02	3.71 (1.95–7.08) ***	3.99 (2.09–7.61) ***
Yes	116	28,675	4.05	115	25,855	4.45	1.10 (0.85–1.42)	1.10 (0.85–1.42)

CI = confidence interval; HR = hazard ratio; IR = incidence rate; PY = person-years. ^†^ Incidence rate per 1000 person-years. ^#^ Adjustment for age, gender, comorbidity, and medication. ^‡^ Participants with any comorbidity were selected into the comorbidity group; * *p* < 0.05, ** *p* < 0.01, *** *p* < 0.001.

**Table 4:** Events and hazard ratios of acute myocardial infarction associated with mean number of annual emergency department visit for participants with pneumoconiosis compared with those without pneumoconiosis.

	Events	Crude HR (95% CI)	Adjusted HR ^†^ (95% CI)
Non-pneumoconiosis	128	1 (reference)	1 (reference)
Pneumoconiosis			
Annual ED visits			
<1	124	1.29 (1.01–1.65) *	1.30 (1.01–1.66) *
≥1	31	1.60 (1.08–2.36) *	1.68 (1.13–2.50) *

CI = confidence interval; ED = emergency department; HR = hazard ratio. ^†^ Adjustment for age, sex, comorbidity, and medication; * *p* < 0.05.

## Data Availability

All data generated or analyzed during this study are included in this manuscript.
